# How does Public Financial Management (PFM) influence health system efficiency: A scoping review

**DOI:** 10.12688/wellcomeopenres.22533.1

**Published:** 2024-10-02

**Authors:** Anita Musiega, Benjamin Tsofa, Edwine Barasa

**Affiliations:** 1 Health Economics Research Unit, KEMRI Wellcome Trust Research Programme, Nairobi, Nairobi, 00100, Kenya; 2 Institute of Healthcare Management, Strathmore University Business School, Strathmore University, Nairobi, Nairobi County, Kenya; 3 Health Systems and Research Ethics Department, KEMRI-Wellcome Trust Research Programme, Kilifi, Kenya; 4 Centre for Tropical Medicine and Global Health, Nuffield department of Medicine, University of Oxford, Oxford, UK

**Keywords:** Public Financial Management, efficiency, health systems

## Abstract

**Background:**

Effective Public Financial Management (PFM) approaches are imperative in the quest for efficiency in health service delivery. Reviews conducted in this area have assessed the impact of PFM approaches on health system efficiency but have left out the mechanisms through which PFM influences efficiency. This scoping review aims to synthesize evidence on the mechanisms by which PFM influences health system efficiency.

**Methods:**

We searched databases of PubMed and Google Scholar and websites of the World Health Organization (WHO), World Bank and Overseas Development Institute (ODI) for peer-reviewed and grey literature articles that provided data on the relationship between PFM and health system efficiency. Three reviewers screened the articles for eligibility with the inclusion criteria. Data on PFM and health system efficiency was charted and summarized. We then reported the mechanisms by which PFM influence efficiency.

**Results:**

PFM processes and structures influence health system efficiency by influencing; the alignment of resources to health system needs, the cost of inputs, the motivation of health workers, and the input mix.

**Conclusion:**

The entire budget process influences health system efficiency. However, most of the findings are drawn from studies that focused on aspects of the budget process. Studies that look at PFM in totality will help explore other cross-cutting issues within sections of the budget cycle; they will also bring out the relationship between the different phases of the budget cycle.

## Plain language summary

Effective management of public finances is crucial for health services, but previous reviews have not fully explained how this happens. This review aims to uncover the ways in which public financial management influences health system performance. By searching medical and financial databases, as well as major organizations' websites, we identified relevant studies and reports that explain this relationship. Our analysis found that PFM processes influence the entire health budget process, from planning to spending, and monitoring, all of which play a significant role in health system performance. However, most studies have focused on specific parts of the budget process. Future research should explore the entire budget cycle to understand how its different stages are interconnected and affect health system performance.

## Introduction

Public funds offer a sustainable and equitable source of financing for the health system ^1^. However, the amount of public funding allocated to healthcare can be limited by economic growth and competing priorities from other sectors ^2^. Improving efficiency in the use of public funds for health offers an avenue for mobilizing additional resources for the health sector that’s within the control of the health system ^3^. Efficiency in healthcare systems refers to the extent to which health system objectives are met with available resources, and there are two types: (1) allocative efficiency which entails maximizing output with the best input combination and (2) technical efficiency which involves getting maximum outputs with available inputs or achieving a given set of outcomes using the least amount of outputs ^4^. Inefficiencies within the health sector result in the wastage of 20–40% of health resources ^5^.

The management of public funds is based on the Public Financial Management (PFM) processes ^6^ which entails the laws, processes, rules, and institutions established by governments to collect, allocate, spend, and account for public funds ^4, 7^. PFM is integrated into the public budget process which entails budget formulation, execution, and monitoring and evaluation ^8^. During budget formulation the government plans and allocates resources to meet its objectives. Budget execution encompasses the provision of promised revenues and the use of these resources to achieve health system objectives. Budget monitoring entails the evaluation of the implementation and achievement of budgetary goals. PFM key objectives are to promote fiscal discipline, allocative efficiency, and technical efficiency ^6^.

PFM has been identified as a determinant of health system efficiency ^9, 10^. These papers have further shown the duality of PFM as both an enabler and a deterrent of health system efficiency ^10, 11^. To gain a deeper understanding of how PFM influences health system efficiency, we conducted a scoping review ^11, 12^. Previous literature reviews have only focused on the relationship between PFM and efficiency, without exploring the mechanisms by which PFM influences efficiency ^13, 14^. Exploring the mechanisms may provide policy levers for improved health system efficiency.

## Methods

We were guided by Arkesy and O’Malley’s methodological framework for scoping reviews ^15^ and the enhancements by Levac ^16^ following the steps 1) Identifying the question 2) Identifying relevant studies 3) Selecting articles and 4) data extraction and synthesis. We used the PRISMA extension ^17^ for scoping reviews to report the findings of the study ^15^. We did not register the protocol for this study in advance.

### Identifying the research question

We sought to answer the question, "How do Public Financial Management (PFM) processes influence health system efficiency?”. The definition of PFM was broad to accommodate aspects of the budget process such as the planning process, priority setting, provider payment mechanisms, procurement processes and auditing, all of which are part of the PFM process. We defined *efficiency* as both technical and allocative. Technical to mean maximizing outputs/outcomes with available resources or minimizing inputs for a given set of outcomes. Allocative to mean the best combination of inputs. The purpose of this scoping review was to identify key concepts about PFM that may influence efficiency and to identify knowledge gaps in the relationship between PFM and health system efficiency ^12^.

### Identifying relevant studies

**Figure 1.**
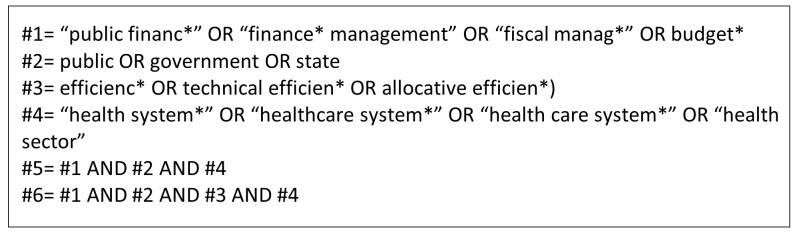
PubMed search.

We searched PubMed and Google Scholar and websites of World Health Organization (WHO), World Bank, Overseas Development Institutes (ODI), and International Budget Partnership (IBP) for relevant articles on PFM and health system efficiency. We only included studies published in English. We used four key search terms, their related synonyms, and combinations to develop a search strategy: “Public” AND “Financial Management” AND “efficiency” AND “health system”. We developed a Boolean Algorithm to search PubMed ( Figure 1). We also searched references of selected papers for other relevant studies. The final search was done in May 2024.

### Selecting articles

**Figure 2.**
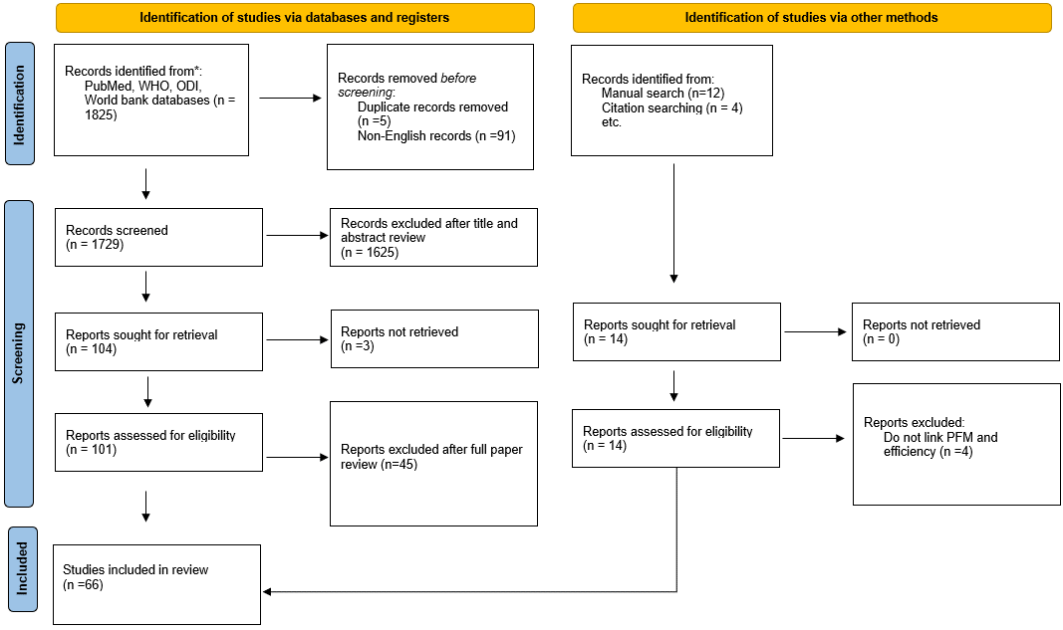
The study selection process flow chart (adapted from PRISMA flow diagrams).

We included articles that focused on the entire PFM subject or an aspect of PFM, such as budget formulation, budget execution and budget monitoring ( Figure 2). We included peer-reviewed empirical studies and grey literature from WHO, World Bank, and ODI but excluded systematic literature reviews. We included studies that 1) Evaluated an aspect of PFM; either budget formulation, execution, or evaluation, or an aspect under the three stages 2) articles that attempted to relate the PFM aspect to health system efficiency. For the second aspect, we first searched the abstract and included only articles that matched this criterion. We excluded studies that 1) Described but did not evaluate an aspect of PFM 2) Did not link PFM to health system efficiency. After de-duplication, we conducted both title and abstract screening. AM and EB reviewed all the titles and abstracts to determine eligibility for full-text review. AM reviewed all full texts as the primary reviewer. EB acted as a secondary reviewer to determine inclusion. Because most of the literature on PFM and efficiency is grey, we did not conduct quality appraisal of selected articles.

### Data extraction and synthesis

We applied the thematic analysis approach to extract and synthesis findings. These followed the steps of familiarization, coding, and charting. During familiarization, we read and reread the articles included in the study. We then applied a data extraction form that we developed from the review question- How PFM influenced health system efficiency. Following familiarization, we coded the data using codes developed inductively and deductively. We then further categorized the codes into four major themes: budget formulation and approval, budget implementation, budget monitoring and evaluation, and actors. Each theme had several sub-themes; for example, under budget formulation, we report five subthemes: budget ceilings, budget structure, alignment of plans and budgets, costing, pooling of funds and priority setting processes. AM finalized the categorization of sub-themes, and where there was uncertainty about the allocation of specific sub-themes, this was resolved by discussions between AM, EB and BT. We then charted the data onto an Excel framework that allowed us to summarize the findings by category, compare between papers and identify linkages.

## Results

The PubMed, Google Scholar, and organizational websites literature search resulted in 1825 items; we removed five duplicates and 91 non-English publications. 1625 items were excluded based on the title and abstract. We did a full paper review for 101 papers and an additional 14 papers added through experts in the field and manually searching references of selected papers. At the full paper review, we excluded 45 records that did not link PFM and service delivery. A total of 66 papers are included in this review (extended data).

### Article overview

**Figure 3.**
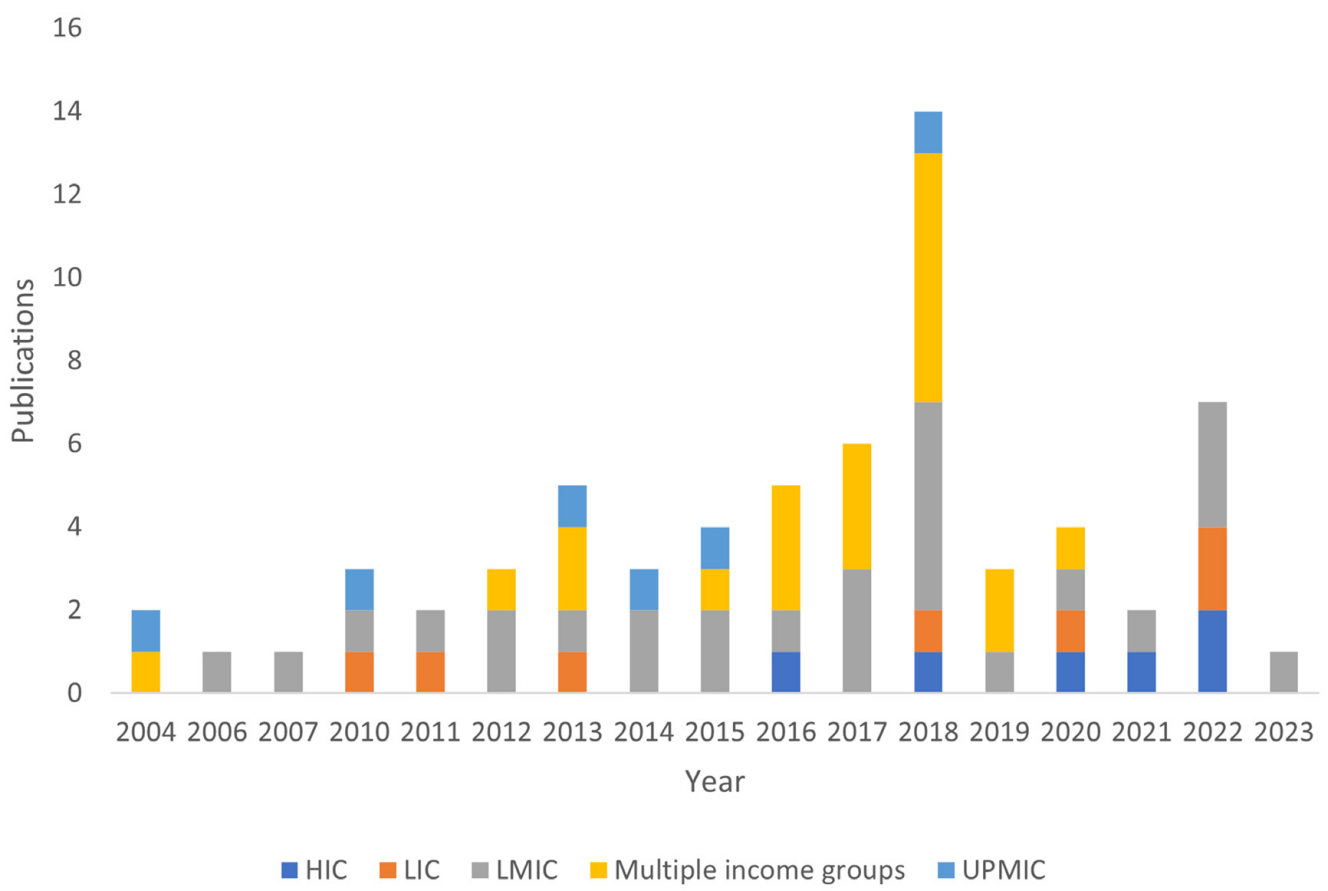
Publications screened by year and country income group.

Of the 66 papers included in this review, 37 are peer reviewed studies while 29 are from grey literature. On the study setting, two are in high income countries (HIC), 44 in low- and middle-income countries (LMIC) and 14 targeted both HIC and LMIC countries ( Figure 3). A total of 10 papers focused on budget formulation only, 14 on budget execution, four budget formulation and execution, six focused on health service delivery and 20 on PFM in general.

***How PFM influences health system efficiency***

The mechanisms through which PFM influences efficiency are explained in the three levels of the budget process: budget formulation, execution, and monitoring.

### Budget formulation and approval

Budget formulation and approval entails projecting expected funds, planning for the funds, and seeking legislative authorization for the plans before execution ^8, 18, 19^. Challenges and opportunities within budget formulation had a ripple effect downstream the PFM process (in budget execution and accountability). The papers reviewed showed that budget formulation structures and processes could potentially influence health system efficiency through the following five aspects; the budget ceiling ^20– 24^, the budget structure ^22, 25, 26^, alignment 0f plans to budget ^23, 27– 29^, costing ^22, 30– 32^, and pooling of the funds ^10, 33– 35^.

**Budget ceilings - the amount of money allocated to the health system – influenced health system efficiency**. There was consensus that adequate and indicative budget ceilings that were allocated based on strategic criteria and issued on time improved service delivery outcomes by improving the alignment of plans to budgets ^21– 23, 36^. For example, in Kenya ^37^ and Uganda ^38^, insufficient budget allocation led to inadequate infrastructure for service delivery and poor service delivery, respectively. Also, in Thailand, historical budget ceilings within the civil servants’ scheme encouraged inefficiency because the more the scheme spent, the more they were allocated ^39^. Finally, ceilings that were not indicative of the actual funds resulted in service delivery interruptions in DRC because budgets were not honoured ^40^, and cash budgeting and replanning in Ghana because of the significant differences between ceiling projections and resource availability ^41^.

**The papers reported use of different budget structures with consequences on efficiency.** The structures used included line budgets ^10, 25, 34 42, 43^ programme based budgets ^10, 30, 36, 44^ and outcome budgets ^45^, with some countries using both functional and economic classification of budgets ^30^. Budget structures that linked resources to priorities and outcomes ^22, 26, 46^ and allowed flexibility in the use of resources enhanced service delivery ^25^ with positive implications on health system efficiency. For example, several reports on PFM for health have shown that the use of line-item budgets disconnected priorities and resources ^25, 46^, resulting in health systems that focused on infrastructural projects rather than purchasing benefits for the population ^40^. Another report on how health financing strategies can support scale-up on NCD interventions noted that input-based budgeting encouraged inefficiency and inequity by encouraging the status quo in resource allocation because efficiency and equity were not considered during the budget formulation processes ^25^.

**The alignment of health budgets with plans had a likely impact on efficiency**. When health systems failed to link available resources to health system plans then they compromised efficiency in two ways. First plans that were not included in the budget were not funded and therefore not implemented ^23, 25, 29, 43, 47, 48^, second the misalignment compromised both financial and performance accountability as at the end of the financial year, the health system could not link the resources used to outcomes produced ^22, 46^. For example, in Lesotho, a study that tested implementation progress for PBB found that the plans and PBB development happened under different structures. As a result, the plans and budgets could not align. The misalignment made it challenging to monitor performance ^22^. A study by Waithaka *et al.* in Kenya that looked at the planning and budgeting processes within counties found that these processes were not aligned; budgets did not incorporate plans which reflected county priorities the plans were therefore not implemented ^28^. Another report indicated that to enhance efficiency, Ethiopia developed one plan, one budget and one report initiative to ensure that plans, budgets and reports are aligned ^35^.

**Pooling of health system resources was associated with clarity and predictability in the budget envelope that enabled effective planning and, possibly enhanced efficiency** ^10, 34, 38, 49^. Besides, pooling allowed for effective resource reallocation, enhanced accountability, and streamlined the incentives for service delivery. For example, in China, the national government used earmarked funding to enhance specific health goals at the local level. However, there was uncertainty about the sustainability of these funds, and second, the study reported reallocation of funds from the health systems' operation budget to other sectors following the introduction of earmarked transfers ^49^. A WHO report on PFM bottlenecks and UHC opportunities noted that in Sierra Leone, multiple fragmented funding sources with different manuals and different bank accounts limited transparency and accountability ^50^.

**Priority setting mechanisms influenced efficiency.** Evidence based priority setting was more likely to result in efficiency ^51^. Countries whose priorities were skewed to curative rather than primary healthcare were more likely to be inefficient. For example, Ethiopia enhance efficiency by investing in high impact primary healthcare interventions ^52^. Kenya on the other hand was deemed inefficient as the focus was more on curative rather than preventive care ^53^.

### Budget execution

Budget execution entailed the release of funds and the use of the funds to finance the provision of health services ^10, 54^; it included several steps; expenditure verification, payment approval and actual payment ^50^. For most African countries, the budget execution phase was the weakest part of the budget cycle; there were risks of losses of resources from the health system in every step of the budget execution ^24, 32^. Budget execution influence efficiency through several ways: credibility of the budget -the extent to which the government honored the approved budget, timeliness of the cash disbursement process, financial controls, financial management systems, fraud and corruption, ring-fencing of funds and provider payment mechanisms.

**Low budget credibility undermined the planning and budgeting phase of the budget**. Because budgets were not honoured, health systems had to re-budget based on the funds received ^41^. Low budget credibility also undermined service delivery as it led to shortage of supplies ^10, 55^. For example, in Ghana, a study that reviewed the budget cycle found that the budget cuts during execution led to development of new budgets based on the amount received; this undermined the budget formulation stage ^41^. Also, in Zambia and Tanzania, a study that linked the budget process to health system performance found that the degree to which the budget was honored was inadequate; this compromised the quality of service delivery by limiting the ability to purchase medical supplies ^10^.

**Cash disbursement delays were linked to health system inefficiency** through 1) interrupted service delivery ^34, 41, 44, 47, 55, 56^ 2) health worker demotivation due to salary delays and lack of resources ^34, 44^ 3) Loss of resources in the process of chasing funds ^44^ 4) increased supplier costs to accommodate cash disbursement delays 5) patient and resource shifting that often led to informal payments which created barriers for access ^57^ 6) compromised tendering processes because the systems lacked a credible platform (good credit history) to contract suppliers ^10, 58^ and ultimately 7) poor budget absorption ^43, 44^. For example, in Yemen, a study that assessed value for money in the health system found that cash disbursement delays had forced many facilities to halt operations and made absenteeism of health workers rampant ^34^. In Zimbabwe, a study that examined the purchasing arrangements for health services found that cash flow challenges undermined service delivery through poor planning, dilapidated infrastructure, poor equipment maintenance, and shortage of supplies and equipment ^47^. In Ghana, a facility manager had to spend half their allocation to cater for transport and accommodation to chase delayed funds at the capital in Accra ^44^.

**Financial controls were associated with both efficiency and inefficiency** ^59^. They could enable service delivery by preventing misappropriation of funds or be a stumbling block by preventing budget execution. Rigid internal controls resulted in cash disbursement delays ^60^, limited autonomy for health workers ^10, 26, 61, 62^ and reduced budget absorption. For example, in Ghana, a study that examined how the untimely release of funds influenced health service delivery found that half of each financial year was lost in financing procedures, and as a result, plans were not implemented ^44^. A WHO report on PFM and UHC noted that in DRC, every stage of controls reduced the funds available to healthcare; of the total budget, only 65% was committed, 55% validated for payment, 50% approved for actual payment, and 40% paid to providers ^32^. In South Africa, strict PFM controls that did not consider the health workers or patient needs resulted in poor patient outcomes ^62^.

**The Financial Management Information Systems (FMIS) were said to influence provider autonomy, the efficiency of cash disbursement, resource fragmentation and accountability**. For example, in Armenia, a study that examined the transition to programme based budgeting reported that the treasury was responsible for budget execution and all payments were made through the treasury financial management system limiting autonomy for health workers ^36^. In Tanzania, a study that linked the budget cycle to performance criteria in health found that investment in a Financial Management system that was simple and easy to use even at the lowest level gave the facilities more autonomy, thereby increasing efficiency ^10^.

**Fraud and corruption were associated with inefficiency** ^50^. They led to loss of resources from the health system. For example, in Mozambique, a WHO reported that misappropriation of resources during budget execution reduced spending on sector priorities ^32^. In South Africa, Folscher and Kruger found high instances of wasteful expenditure that resulted in overall poor performance and bad audit reports ^29^. Another report by the WHO on public financing for health in Africa noted that improving the budget envelope's actualization depends partly on reducing leakages that resulted from a deficiency in healthcare financial management ^61^.

**Failure to ring-fence health sector funds was associated with loss of resources to other sectors.** This reduced healthcare resources undermining service delivery and health output **s** ^24^. For example, in DRC, a WHO report noted that health funds were not ring-fenced and were used for other administrative activities under the president's office, reducing the budget available for health ^24^. In Kenya, a study by Waithaka *et al.*, found that funds that were planned for purchasing motorbikes for the health system were reallocated to another governors projects which he had promised the public ^28^

**Strategic provider payment mechanisms enhanced efficiency.** Provider payment mechanisms influenced provider behavior thereby influencing efficiency. Mixed provider payment mechanisms led to patient and resource shifting with likely consequences on efficiency ^57, 63^. For example, in Kenya, line item budget did not incentivize providers to strive for efficiency, instead the providers charged the patient additional fees as they felt that the funds were not sufficient ^64^. Also in Nigeria, mixed provider payment mechanisms led to resource shifting from funding with rigorous accountability mechanisms to those with less rigorous accountability mechanisms ^57^.

### Budget monitoring and evaluation

Budget monitoring and evaluation influenced health service delivery through three main mechanisms, first the fragmented accountability channels. Disconnected accountability for finances and service delivery undermined the synergy required to achieve health service delivery ^56^. Piatti *et al.* found that budget evaluation in Zambia and Tanzania was compliance-driven with inadequate attention to health system goals of efficiency, equity, and quality ^10^. Also, in Ghana, there were conflicting reporting channels to the district and the Ghana Health Services in Accra, which compromised accountability from department of health ^41^. Finally, in Nigeria, multiple funding flows led to shifting of resources and patients from areas with complex accountability to those that required little accountability ^57^.

**Also, the papers found that social accountability was critical in enhancing health outcomes and likely efficiency**. For example, in China, Brixi *et al.* found that sub-national governments had insufficient downward accountability; thus, the health sector performed poorly compared to the agricultural sector, where there were established mechanisms for downward accountability ^49^. In Kenya, a facility with limited community support as the community health committee was inactive did not achieve the desired targets ^65^, unlike those with community support. Also, In Kenya, the implementation of plans and budgets was unsatisfactory because the hospitals lacked internal accountability mechanisms to follow up and ensure plans and budgets were implemented ^27^. In Thailand, the UCS scheme had a Facebook page and 24-hour call centre to provide clients with information and resolve disputes. As a result, it was owned by the people; this was thought to contribute to its success and efficiency ^39^. Besides, weak accountability had a direct impact over the use of public funds to deliver services ^29, 65^.

**Both supply-side and demand-side incentives influenced the utilization and cost of services, both of which influenced efficiency** ^40, 66^. Health worker incentives and sanctions for excellent and poor performance significantly influenced efficiency ^2, 60^. For example, in Romania, adverse audit reports led to minimal changes as the sanctions and rewards were not enforced. Instead, hospitals with higher debts received more attention from both government and NGOs and sometimes more financing. As a result, hospitals intentionally got into debt resulting in inefficiency ^21^. In South Africa, the Eastern Cape department was subjected to several government interventions for non-performance and poor audit reports for 12 years with little or no change ^29^. In 2010 following sanctions for malfeasance around public finance, the eastern cape department recorded a significant improvement in service delivery and decreased wastage of resources.

### Actors

**Stakeholder involvement in the budget process had an influence on health system efficiency**. Community-level stakeholder involvement in the PFM process increased awareness of services, demand for services and accountability. Communities that were more aware were better placed to demand accountability ^67^. For example, in Kenya, a pilot study that tested the integration of social accountability mechanisms in health service delivery found that acceptance and support from the community increased demand for services ^65^. The study also found that facilities with better support from the community were more likely to meet their service delivery targets and were, therefore, more efficient ^65^.

Frontline worker stakeholder involvement in the budget process enhanced alignment of plans to budget, motivated health workers and increased mobilisation of funds for the health sector. For example, in Kenya, a study that examined hospital autonomy in the context of devolution found that decreased stakeholder involvement was associated with misalignment of plans and budgets and failure to implement planned activities ^68^. In the Philippines, frontline worker management of the budget process increased funding from other sources such as the private sector and user fees ^69^.

**The success of the PFM arrangements in transforming budgets to health system outcomes depended on the actors in the process** ^60^. For example, in Ghana, the external audit was the responsibility of the auditor general. Late and poor audit reports from their office meant that the accountability mechanisms for the health system were poor ^60^. According to Duran et al, there was widespread distrust among the hospital management team, the hospital board was seen as a bureaucratic addition that did not facilitate the budgeting process ^21^. In South Africa, the report by Folshcer and Kruger reported an undue influence of unions over hospital mangers that perpetuated indiscipline and poor work ethics ^29^. Also in South Africa, according to Duran et al changes in the leadership positions led to significant changes in the performance of the health system, with improved financial management and accountability ^29^.

## Discussion

This review sought to synthesize evidence on how PFM influences health system efficiency. While PFM has been identified as a determinant of efficiency, few studies have examined the relationship between PFM and efficiency of health systems. This gap is concerning as efficiency gains have been identified as one of the crucial sources of domestic fiscal space for health, and PFM is a determinant of efficiency. For health systems to actualize PFM related efficiency gains, it is vital to understand how PFM structures and processes influence efficiency. These are likely to provide policy levers for increased fiscal space for health. Besides, most of the studies that relate to PFM and efficiency have been piecemeal, considering singular aspects of PFM such as planning, budgeting, priority setting, provider payment mechanisms, rather than PFM in its totality. While these studies have drawn essential lessons on each aspect, it is vital to understand how all these issues are interrelated and how their interaction influences overall system efficiency.

Nonetheless, from the papers included in the review, we draw several mechanisms through which PFM influences health system efficiency. PFM processes and structures influence health system efficiency by influencing; the alignment of resources to needs, the cost of inputs, the motivation of health workers, and the input mix.

The alignment of plans and budgets ensures that limited health system resources are allocated to where they are most needed and where they will have the greatest impact on health outcomes and therefore efficiency ^23^. From this review, PFM processes negatively influence this alignment in several ways: use informal priority setting mechanisms ^51, 70, 71^, use of historical ceilings, failure to avail ceilings, use of line-item budgets, fragmented budgeting, failure to ring fence health funds and limited stakeholder involvement in planning and budgeting.

The cost of health system goods and services directly impacts efficiency, with more expensive goods and services leading to greater inefficiency ^72^. From the literature review, the PFM processes influence the cost of inputs and therefore efficiency in various ways: cash disbursement delays led to a poor credit history and therefore increased cost of supplies, fraud and corruption led to loss of resources from the health sector and therefore increased expenditure against limited outputs, and fragmented accountability encouraged the misappropriation of funds.

The motivation and productivity of health workers also impact on health system. PFM processes and structures influenced health worker motivation and productivity in various ways. Cash disbursement delays led to salary delays that demotivated employee. Excessive controls limited provider autonomy that demotivated employees reducing their productivity, passive provider payment mechanisms failed to encourage productivity.

An adequate input mix is important for health service delivery and therefore health system efficiency ^72^. From the review, PFM processes influence the input mix and therefore efficiency in various ways; inadequate ceilings limit the resources available for planning and budgeting, non-indicative ceilings or limited budget credibility leads to an inappropriate input mix that limits health service delivery, rigid financial controls lead to delayed release of funds for certain activities leading to an inappropriate input mix, and finally failure to ring fence health funds exposes them to reallocation that has the same impact as an inadequate/non-indicative ceiling.

## Limitations

One limitation of this review is that we only included articles published in English. Second, most of the studies included did not focus on PFM in totality rather an aspect of PFM, this may have affected the depths of our discussions. Third, the review includes grey literature, however with the vast amount of grey literature, the selected articles may not be exhaustive. These may have implications on the findings and the discussions.

## Conclusions

Despite the limitations, PFM structures and processes have potential effects on health system efficiency. However, for low and middle countries to actualize efficiency gains, it is important for them to understand how PFM and why the various aspects of PFM influence efficiency.

## Ethics and consent

This study received ethics approval from the KEMRI Scientific and Ethics Review Unit (SERU), approval number SERU/CGMR-C/154/3814 first approved on 22/03/2019 and subsequently renewed on 04/05/2020 and 04/05/2021. Consent was not required as this study summarizes existing publicly available literature.

## Data Availability

All data underlying the results are available as part of the article and no additional source data are required. Harvard Dataverse: How does Public Financial Management (PFM) influence health system efficiency: A scoping review. https://doi.org/10.7910/DVN/9SZZGV ^17^. This project contains the following extended data: - Characteristics of studies included in the review.docx - DataReadme_Musiega_et_al_review.txt (description of the project and the data) Harvard Dataverse: PRISMA-ScR checklist for ‘How does Public Financial Management (PFM) influence health system efficiency: A scoping review https://doi.org/10.7910/DVN/9SZZGV ^17^. Data are available under the terms of the Creative Commons Attribution 4.0 International license (CC-BY 4.0).

## References

[1] Brundtland GH : Public financing for primary health care is the key to universal health coverage and strengthening health security. *Lancet Glob Health.* 2022;10(5):e602–e603. 10.1016/S2214-109X(22)00166-8 35390346 PMC9838789

[2] Barroy H Sparkes S Dale E : Can Low-and Middle-Income Countries increase Domestic Fiscal Space for Health: a mixed-methods approach to assess possible sources of expansion. *Health Syst Reform.* 2018;4(3):214–226. 10.1080/23288604.2018.1441620 30081685

[3] Barroy H Sparkes S Dale E : Assessing fiscal space for health in Low and Middle Income Countries: a review of the evidence. WHO. no. 3. (WHO/HIS/HGF/ HFWorkingPaper/16.3,2016 Reference Source

[4] Allen R Hemming R Potter BH : The international handbook of Public Financial Management.2013;66. 10.1057/9781137315304

[5] World Health Organization: The world health report: health systems financing: the path to universal coverage.2010; Accessed: Jan. 30, 2023. Reference Source 10.2471/BLT.10.078741 PMC2878164 20539847

[6] Schick A : A contemporary approach to public expenditure management.1998. Reference Source

[7] Andrews M Cangiano M Cole N : This is PFM working papers. no. 285.2014. Reference Source

[8] World Health Organization: Aligning Public Financial Management and health financing: a process guide for identifying issues and fostering dialogue. Geneva,2017. Reference Source

[9] Zeng W Yao Y Barroy H : Improving fiscal space for health from the perspective of efficiency in Low- and Middle-Income Countries: what is the evidence? *J Glob Health.* 2020;10(2):1–10, 020421. 10.7189/jogh.10.020421 33110580 PMC7568933

[10] Piatti-Fünfkirchen M Schneider P : From stumbling block to enabler: the role of Public Financial Management in health service delivery in Tanzania and Zambia. *Health Syst Reform.* 2018;4(4):336–345. 10.1080/23288604.2018.1513266 30398392

[11] Zeng W Musiega A Oyasi J : Understanding the performance of county health service delivery in Kenya: a mixed-method analysis. *Health Policy Plan.* 2022;37(2):189–199. 10.1093/heapol/czab129 34718555 PMC7613432

[12] Munn Z Peters MDJ Stern C : Systematic review or scoping review? Guidance for authors when choosing between a systematic or scoping review approach. *BMC Med Res Methodol.* 2018;18(1):1–7, 143. 10.1186/s12874-018-0611-x 30453902 PMC6245623

[13] Goryakin Y Revill P Mirelman AJ : Public Financial Management and health service delivery: a literature review.2020;191–215. 10.1142/9789813272378_0007

[14] Welham B Hart T Mustapha S : Public Financial Management and health service delivery necessary, but not sufficient? 2017; Accessed: Oct. 26, 2019. Reference Source

[15] Tricco AC Lillie E Zarin W : PRISMA extension for Scoping Reviews (PRISMA-ScR): checklist and explanation. *Ann Intern Med.* 2018;169(7):467–473. 10.7326/M18-0850 30178033

[16] Levac D Colquhoun H O'Brien KK : Scoping studies: advancing the methodology. *Implement Sci.* 2010;5:1–9, 69. 10.1186/1748-5908-5-69 20854677 PMC2954944

[17] Musiega A Tsofa B Barasa E : How does Public Financial Management (PFM) influence health system efficiency: a scoping review. [Dataset].2024. 10.7910/DVN/9SZZGV PMC11502999 39464374

[18] Simson R Sharma N Aziz I : A guide to Public Financial Management literature: for practitioners in developing countries. ODI publication, December,2011;1–39. Reference Source

[19] World Health Organization: Public Finance Management within health financing.2017;2:2–5. Reference Source

[20] Kiross GT Chojenta C Barker D : The effects of health expenditure on infant mortality in sub-Saharan Africa: evidence from panel data analysis. *Health Econ Rev.* 2020;10(1):1–9, 5. 10.1186/s13561-020-00262-3 32144576 PMC7060592

[21] Duran A Chanturidze T Gheorghe A : Assessment of public hospital governance in Romania: lessons from 10 case studies. *Int J Health Policy Manag.* 2019;8(4):199–210. 10.15171/ijhpm.2018.120 31050965 PMC6499904

[22] Vian T Bicknell WJ : Good governance and budget reform in Lesotho public hospitals: performance, root causes and reality. *Health Policy Plan.* 2014;29(6):673–684. 10.1093/heapol/czs121 23293099

[23] Tsofa B Molyneux S Goodman C : Health sector operational planning and budgeting processes in Kenya-"never the twain shall meet". *Int J Health Plann Manage.* 2016;31(3):260–276. 10.1002/hpm.2286 25783862 PMC4988384

[24] Le Gargasson JB Mibulumukini B Gessner BD : Budget process bottlenecks for immunization financing in the Democratic Republic of the Congo (DRC). *Vaccine.* 2014;32(9):1036–1042. 10.1016/j.vaccine.2013.12.036 24384055

[25] Jakab M Evetovits T McDaid D : Health financing strategies to support scale-up of core noncommunicable disease interventions and services.In: *Health Systems Respond to Noncommunicable Diseases: Time for Ambition.*M. Jakab, J. Farrington, L. Borgermans, and F. Mantingh, Eds., Copenhagen, Denmark: WHO regional Office for Europe,2018;200–223. Reference Source

[26] Barroy H Andre F Nitiema A : Transition to programme budgeting in health in Burkina Faso. 11. Reference Source

[27] Barasa EW Cleary S Molyneux S : Setting healthcare priorities: a description and evaluation of the budgeting and planning process in county hospitals in Kenya. *Health Policy Plan.* 2017;32(3):329–337. 10.1093/heapol/czw132 27679522 PMC5362066

[28] Waithaka D Tsofa B Kabia E : Describing and evaluating healthcare priority setting practices at the county level in Kenya. *Int J Health Plann Manage.* 2018;33(3):e733–e750. 10.1002/hpm.2527 29658138 PMC6120533

[29] Folscher A Kruger J : When opportunity beckons: the impact of the public service accountability monitor’s work on improving health budgets in South Africa. *SSRN Electron J.* 2013. 10.2139/ssrn.2326587

[30] Nnaji GA Oguoma C Nnaji LI : The challenges of budgeting in a newly introduced district health system: a case study. *Glob Public Health.* 2010;5(1):87–101. 10.1080/17441690903371762 19946811

[31] World Health Organization: WHO symposium on health financing for UHC public financing for UHC : towards implementation.Montreux, Switzerland.2018. Licence: CC BY-NC-SA 3.0 IGO.

[32] World Health Organization [WHO]: Leveraging Public Financial Management for better health in Africa. Geneva,2019.

[33] Smith PC Witter S : Risk pooling in health care financing: the implications for health system performance.Washington, D.C.2004. Reference Source

[34] Elgazzar HA : Raising returns: the distribution of health financing and outcomes in Yemen.2011. Reference Source

[35] Asfaw A Bharali I Glenday G : Public Financial Management perspectives on health sector financing and resource allocation in Ethiopia. *SSRN Electron J.* 2020. 10.2139/ssrn.3534342

[36] Dale E Kyurumya A Kharazyan S : Budget structure reforms and transition to programme budgeting in health: lessons from Armenia.2018;12:39. Reference Source

[37] Nyawira L Mbau R Jemutai J : Examining health sector stakeholder perceptions on the efficiency of county health systems in Kenya.2020. 10.1371/journal.pgph.0000077 PMC10021822 36962100

[38] Musango L Orem JN Elovainio R : Moving from ideas to action - developing health financing systems towards universal coverage in Africa. *BMC Int Health Hum Rights.* 2012;12(1):1–11, 30. 10.1186/1472-698X-12-30 23137065 PMC3558372

[39] Patcharanarumol W Panichkriangkrai W Sommanuttaweechai A : Strategic purchasing and health system efficiency: a comparison of two financing schemes in Thailand. *PLoS One.* 2018;13(4): e0195179. 10.1371/journal.pone.0195179 29608610 PMC5880375

[40] World Health Organization: Fiscal space, public financial management, and health financing: sustaining progress towards UHC. Implementation of the Collaborative Agenda, 26–28 April, 2016. Reference Source

[41] Abekah-Nkrumah G Dinklo T Abor J : Financing the health sector in Ghana: a review of the budgetary process. *European Journal of Economics, Finance and Administrative Sciences.* 2009; (17):45–59. Reference Source

[42] Afzali HHA Moss JR Mahmood MA : Exploring health professionals’ perspectives on factors affecting Iranian hospital efficiency and suggestions for improvement. *Int J Health Plann Manage.* 2011;26(1):e17–29. 10.1002/hpm.1035 20603856

[43] Glenngård AH Maina TM : Reversing the trend of weak policy implementation in the Kenyan health sector? – A study of budget allocation and spending of health resources versus set priorities. *Health Res Policy Syst.* 2007;5: 3. 10.1186/1478-4505-5-3 17394640 PMC1851957

[44] Asante AD Zwi AB Ho MT : Getting by on credit: how district health managers in Ghana cope with the untimely release of funds. *BMC Health Serv Res.* 2006;6: 105. 10.1186/1472-6963-6-105 16916445 PMC1563463

[45] Rasivhetshele RE Govender KK : Public health funding and health service delivery-a case study of the Gauteng Province, South Africa. *Arch Bus Res.* 2014;2(3):50–61. 10.14738/abr.23.283

[46] World Health Organisation: Fiscal space, Public Finance Management and health financing a collaborative agenda.2014;978:92–4.

[47] Gwati G : Report of field survey of current purchasing practices for district health services in Zimbabwe.2015. Reference Source

[48] Moradi T Kabir MJ Pourasghari H : Challenges of budgeting and Public Financial Management in Iran’s health system: a qualitative study. *Med J Islam Repub Iran.* 2023;37(1): 80. 10.47176/mjiri.37.80 37600636 PMC10439693

[49] Brixi H Mu Y Targa B : Engaging sub-national governments in addressing health equities: challenges and opportunities in China’s health system reform. *Health Policy Plan.* 2013;28(8):809–824. 10.1093/heapol/czs120 23221008

[50] World Health Organisation: Building strong public financial management systems towards Universal Health Coverage : key bottlenecks and lessons learnt from country. Geneva,2018.

[51] Barasa E Molyneux S English M : Hospitals as complex adaptive systems: a case study of factors influencing priority setting practices at the hospital level in Kenya. *Soc Sci Med.* 2017;174:104–112. 10.1016/j.socscimed.2016.12.026 28024239 PMC5267634

[52] Alebachew A Bharali I Glenday G : Public financial management perspectives on health sector financing and resource allocation in Ethiopia. Reference Source

[53] National AIDS Control Council, Government of Kenya (GoK), and World Bank: Laying the foundation for a robust health care system in Kenya. Nascop, Ministry of Health, Government of Kenya,2014;37. Reference Source

[54] WHO | Aligning Public Financial Management (PFM) and health financing. WHO,2017. Reference Source

[55] Mbau R Barasa E Munge K : A critical analysis of health care purchasing arrangements in Kenya: a case study of the county departments of health. *Int J Health Plann Manage.* 2018;33(4):1159–1177. 10.1002/hpm.2604 30074642 PMC6492197

[56] Nxumalo N Gilson L Goudge J : Accountability mechanisms and the value of relationships: experiences of front-line managers at subnational level in Kenya and South Africa. *BMJ Glob Health.* 2018;3(4): e000842. 10.1136/bmjgh-2018-000842 30002921 PMC6038841

[57] Onwujekwe O Mbachu C Ezenwaka U : Characteristics and effects of multiple and mixed funding flows to public healthcare facilities on financing outcomes: a case study from Nigeria. *Front Public Health.* 2020;7:403. 10.3389/fpubh.2019.00403 32010658 PMC6974794

[58] Jowett M Kutzin J, World Health Organziation : Raising revenues for health in support of UHC: strategic issues for policy makers.2015. Reference Source

[59] Moritz Piatti-Fünfkirchen Collins Chansa Dominic Nkhoma.2020; Accessed: May 22, 2024.

[60] Abekah-Nkrumah G Nomo P : In pursuit of a technical need or political compromise: reforms of public financial management practices in Ghana’s Health Sector. *Finance and Development in Africa.* 2015;31:149–169. 10.1108/S1479-3563(2012)000012B011

[61] World Health Organization: Public financing for health in Africa: from Abuja to the SDGs. Geneva, Switzerland,2016. Reference Source

[62] Penn-Kekana L Blaauw D Schneider H : It makes me want to run away to Saudi Arabia’: management and implementation challenges for public financing reforms from a maternity ward perspective. *Health Policy Plan.* 2004;19 Suppl 1:i71–i77. 10.1093/heapol/czh047 15452017

[63] Ezenduka C Obikeze E Uzochukwu B : Examining healthcare purchasing arrangements for strategic purchasing in Nigeria: a case study of the Imo state healthcare system. *Health Res Policy Syst.* 2022;20(1): 41. 10.1186/s12961-022-00844-z 35436965 PMC9013978

[64] Mbau R Barasa E Munge K : A critical analysis of healthcare purchasing arrangements in Kenya: a case study of the county departments of health. *Int J Health Plann Manage.* 2018;33(4):1159–1177. 10.1002/hpm.2604 30074642 PMC6492197

[65] Machira YW Nizam R : Integrating social accountability in healthcare. Delivery: Lessons Drawn from Kenya,2015. Reference Source

[66] World Bank: Fixing the public hospital system in China.2010. Reference Source

[67] Transparency International Česká republika: Analysis of public procurement in the health sector. no.12,2016. Accessed: Apr. 21, 2023. Reference Source

[68] Barasa E Manyara A Molyneux S : Recentralization within decentralization: county hospital autonomy under devolution in Kenya. *PLoS One.* 2017;12(8): e0182440. 10.1371/journal.pone.0182440 28771558 PMC5542634

[69] Liwanag HJ Wyss K : What conditions enable decentralization to improve the health system? Qualitative analysis of perspectives on decision space after 25 years of devolution in the Philippines. *PLoS One.* 2018;13(11): e0206809. 10.1371/journal.pone.0206809 30395625 PMC6218067

[70] Waithaka D Tsofa B Kabia E : Describing and evaluating healthcare priority setting practices at the county level in Kenya. *Int J Health Plann Manage.* 2018;33(3):e733–e750. 10.1002/hpm.2527 29658138 PMC6120533

[71] McCollum R Theobald S Otiso L : Priority setting for health in the context of devolution in Kenya: implications for health equity and community-based primary care. *Health Policy Plan.* 2018;33(6):729–742. 10.1093/heapol/czy043 29846599 PMC6005116

[72] Cylus J Papanicolas I Smith PC : A framework for thinking about health system efficiency.2016; Accessed: Apr. 21, 2023. Reference Source

