# Supplementary material for: How does Public Financial Management (PFM) influence health system efficiency: A scoping review
Source: Wellcome Open Res. 2024 Oct 2;9:566. [Version 1] doi: 10.12688/wellcomeopenres.22533.1 (PMC11502999; doi:10.12688/wellcomeopenres.22533.1)
Supplement: Peer review history

# Reviewer report: Reviewer response for version 1

**DOI:** 10.21956/wellcomeopenres.24828.r102739

**Published:** 2024-10-24

**Authors:** Brendan Kwesiga

**Affiliations:** 1 UNICEF, Nairobi, Kenya

The authors could consider the following comments in the revision.

 From the the studies selection process flow chart it is not clear how you arrived at 66. Does it mean that there was no addition in terms of studies identified from other methods? The total of 66 is 101 minus 45. Were there no additional studies from the other sources reviewed?

 The authors could categorize the studies by the methods they used to show the mechanisms through which PFM influences efficiency.

 The quote below which is made with reference to ***Pooling of health system resources was associated with clarity and predictability in the budget envelope that enabled effective planning and, possibly enhanced efficiency *** is confusing and does not talk directly to how pooling would improve efficiency. Does earmarking of health funds explicitly imply that these will not be held or managed as a pool?  Authors also need to explain how fungibility of funds (funds being reallocated following earmarks) affected efficiency.  

 “For example, in China, the national government used earmarked funding to enhance specific health goals at the local level. However, there was uncertainty about the sustainability of these funds, and second, the study reported reallocation of funds from the health systems' operation budget to other sectors following the introduction of earmarked transfers ^49^. ”

 Under the budget execution section, the authors note that failure to **ring-fence health sector funds was associated with loss of resources to other sectors as** a potential source of inefficiency. This is probably where the earmarking in China could be discussed for you to indicate both the negative and positive of ringfencing/earmarking of funds for the health sector.

**“Both supply-side and demand-side incentives influenced the utilization and cost of services, both of which influenced efficiency”.** I do not think this is a budget monitoring issue. It probably belongs under execution.

 As part of budget monitoring and evaluation, I was expecting issues on the misalignment between programmatic and financial monitoring and accountability and also usefulness of budget classification/charter of account

 With regards to the findings on budget ceilings, did any studies report on the effect of medium-term budget and expenditure frameworks (or maybe broadly mechanism for ensuring predictability of budgets) as influencing efficiency?

 I believe some aspects such as **Fraud and corruption were** associated with inefficiency are not specific to a single component of the budget cycle but could be cross cutting. Since authors group the findings along the budget cycle, they could create on cross cutting category to capture the cross-cutting aspects identified as influencing efficiency. This could also include the influence/effect of mechanisms of external accountability. I do not see any findings related to these (e.g civil society platforms)

Are the rationale for, and objectives of, the Systematic Review clearly stated?

Yes

Is the statistical analysis and its interpretation appropriate?

Not applicable

If this is a Living Systematic Review, is the ‘living’ method appropriate and is the search schedule clearly defined and justified? (‘Living Systematic Review’ or a variation of this term should be included in the title.)

Not applicable

Are sufficient details of the methods and analysis provided to allow replication by others?

Yes

Are the conclusions drawn adequately supported by the results presented in the review?

Yes

Reviewer Expertise:

health economics

I confirm that I have read this submission and believe that I have an appropriate level of expertise to confirm that it is of an acceptable scientific standard.

# Reviewer report: Reviewer response for version 1

**DOI:** 10.21956/wellcomeopenres.24828.r102740

**Published:** 2024-10-22

**Authors:** Mary Ejang

**Affiliations:** 1 Lira University, Lira, Northern Region, Uganda

The article is very instrumental in guiding alignment of resources in the health system. The rationale is partly stated because does not include policy formulation and planning in the health sector.

 The Public Choice theory is relevant in analyzing the Public Finance Management in Health Sector but was excluded. The theory illuminates the roles of key stakeholders in the private and public spheres in managing public resources.

Are the rationale for, and objectives of, the Systematic Review clearly stated?

Partly

Is the statistical analysis and its interpretation appropriate?

Not applicable

If this is a Living Systematic Review, is the ‘living’ method appropriate and is the search schedule clearly defined and justified? (‘Living Systematic Review’ or a variation of this term should be included in the title.)

Yes

Are sufficient details of the methods and analysis provided to allow replication by others?

Yes

Are the conclusions drawn adequately supported by the results presented in the review?

Yes

Reviewer Expertise:

Finance and Accounting

I confirm that I have read this submission and believe that I have an appropriate level of expertise to confirm that it is of an acceptable scientific standard.
